# Some of the Causes Leading to and Producing Suppuration1Read before the section on surgery, Buffalo Academy of Medicine, January 6, 1903.

**Published:** 1903-03

**Authors:** Francis W. McGuire

**Affiliations:** Buffalo, N. Y., 1540 Main Street.


### Some of the Causes Leading to and Producing Suppuration.[1]

By FRANCIS W. McGUIRE, M. D., Buffalo, N. Y.

WOUND infection is a condition which has existed since the beginning of operative surgery. Previous to the days of antiseptics infection was almost a universal rule, but even today it occurs occasionally with all our improved technic. Since the time of Lister there has been a continuous volley fired at the microbe and in our endeavor to completely eradicate this thorn from the side of surgery, we have too frequently neglected other important measures. It is not my object to detract in any way from the importance of our present methods of asepsis, but, on the contrary, I would like to point out a few glaring evidences of defective technic which occasionally are causes of wound infection.

Regarding many of the points I make no claim for originality. While the attention of the surgical world has been called to these conditions on numerous occasions yet their frequent occurrence seems to justify still further emphasis.

One of the first defects with which one is confronted is improper plumbing. The basins are so constructed that in order to bathe the hands with any degree of comfort one is compelled to wash in standing water, one of the most filthy habits. Running sterile water is the ideal arrangement for washing the hands and consequently the plumbing should be so arranged that first the water is controlled by the foot; second, the hot and cold water should come through a common faucet; and, third, this faucet should be placed a sufficient distance from both the wall and basin that hands and arms need not come in contact with either.

Some surgeons seem to be imbued with a false sense of security in the use of antiseptics. It has been observed frequently that after the reinfection of hands during an operation, the surgeon will simply dip them in the antiseptic solution and his surgical conscience is immediately relieved, believing that he has destroyed any germs that may have been lodged on hands or arms. This method cannot be too severely condemned and I have no doubt if the antiseptic solutions were discarded from our operating rooms, we would have cleaner hands by having more effective mechanical cleansing.

1. Read before the section on surgery, Buffalo Academy of Medicine, January 6, 1903.

Next, notice the soap and brushes. Here you meet with a condition that is frequently disgusting, and one feels that it would be better to leave the patient to the ravages of disease rather than subject him to an operation under such conditions. The soap is not sterile, and is placed in vessels so constructed that it is impossible to extract it without coming in contact with the dirty edge of its container. The brushes may have been placed in some antiseptic solution, but are seldom sterilised. Frequently they are of the poorest quality, and, as a rule, have been used so long that there are scarcely any bristles left with which to do effective scrubbing. Assuming that we should introduce the least possible amount of septic material into our operating room, I believe we should not wear our street clothing. Street shoes should be changed for those which can be easily cleansed, and the trousers for those that can be sterilized, both of which can be readily obtained. A sterile long-sleeved gown should be worn, changed when infected, or at least after each operation.

The assistants and nurses should be of the least possible number, as the fewer the hands that come in contact with an operation the better. Two sterile nurses are ample, and possibly one is sufficient if the surgeon has two assistants.

Catgut is a material that is very frequently misused. It should be placed in wide-mouth jars, removed with a clamp, and in no case should it be returned to the jar when once removed, as there is a great probability of its becoming infected. The technic of handling catgut I will not discuss, but will simply say that it should be handled as little as possible. Literature, domestic and foreign, has been prolific regarding the sterilisation of catgut, but, in my opinion, less trouble would accrue from its use if greater care were taken in handling it after its sterilisation. Regarding sterilisation, other things being equal, the greater the degree of heat the better the method. Considered in this light, cumol seems to answer the requirements better than any other method. Another good way to prepare catgut is by the formalin method. This method is simpler than the cumol, as the latter method requires a special steriliser. I have seen a great deal of this preparation used with entire satisfaction.

Instruments that have become infected from any cause should be discarded immediately. This rule seems to be occasionally forgotten, especially when infected from the intestinal canal in such operations as intestinal anastomosis, appendectomies, and the like. Basins that are used in operations should be sterilised by heat. We are not sure of their being sterile when simply washed with a cloth dipped in bichlorid solution, as is the custom in some institutions.

Wounds should be handled as little as possible. The self-retaining retractor is an instrument that should be used more frequently, especially in abdominal work. It prevents handling the wound, protects its edges and holds it open to greater advantage than can an assistant. The ungloved hand must be a prolific source of wound infection. Today it is known that there is no method of rendering the hands sterile. The surgeon will take all manner of trouble to render his instruments, dressings and ligatures sterile, but will without hesitation use his uncovered hand in the wound. Acting according to our present knowledge, it must appeal to everyone that the only positive method of having hands noninfectious is to cover them with a pair of sterile rubber gloves.

The protection of the operating field by towels is unsatisfactory and not frequently do we find them misplaced some distance from their original position, thereby exposing an infected surface. The better cover seems to me to be that by an apron with a small hole, which cannot be displaced. The hole can be easily enlarged to meet existing circumstances.

The part of my paper that I wish to emphasise more especially is the increasing of the resistance of the patient. Bouchard has told us that "physicians ought not to permit themselves to be occupied alone with the research after microbes, but that they ought to busy themselves as well with investigating the circumstances which disarm the organism against microbic invasion." Lawson Tait's method of operating has taught us that by conserving the natural resisting forces of the patient he could almost do away with antiseptic methods.

Autointoxication is of special importance to the surgeon, because the infectious organisms find tissues depressed by the absorption of excrementitious principles, much more vulnerable than those of the healthy individuals. It is important that the bowels should be in a state of activity, in fact they should be as nearly empty as possible, since the loss of a moderate amount of blood and the frequent inability of the stomach to retain water, make absorption of the fluids from the intestines likely to occur. Before undertaking surgical procedures the condition of the heart and lungs should be ascertained, for in certain organic diseases these organs, by the additional burdens of an operation, may cause an autointoxication which, if not dangerous in itself, may become so by favoring local or general infection.

The liver and skin should not be overlooked in preparing a patient. The subject of autointoxication has been too commonly left to the domain of general medicine, and one in which the surgeon as such need take only a passing interest. Park has well said that:

> The surgeon will prove himself the best master of the situation who is thoroughly conversant with all that the general topic of autoinfection comprises and implies, for he will find that his surgical patients will do well or badly just in proportion as he maintains the equilibrium between ingestion and egestion, or as he realises that retained excrementitious products are among the most active predisposing causes of what may be a little later a distinct surgical sepsis.

For a moment let us digress and see what can be done toward increasing the patient's resistance to infection. Where time and circumstances permit, the patient ought to be under treatment at least four days, and in some cases even a longer period of time could be used to advantage. During this period the patient should not indulge in any dissipation; he should have strychnin three or four times a day and sufficient cathartics to keep the bowels moving twice a day. About six hours before operation he should receive an enema and bowel wash. The quantity and quality of urine should be ascertained. He should drink two quarts of water every twenty-four hours and receive a sweat bath every second or third day, followed by a hot bath. The stomach should not receive any food for at least six hours previous to operation.

In preserving the patient's resisting power, I believe there are a few things that compare with rapid operating; almost all patients are in good condition no matter what the operation is, at the end of fifteen minutes of anesthesia, and almost no patient is in a good condition at the end of an hour and a half or two hours. Morris has said that "if work is done in fifteen minutes the operation is executed, but if in an hour and fifteen minutes, perhaps it is the patient that is executed."

The anesthetic and the manner of administration has an important bearing. The commencing of an anesthetic by nitrogen monoxide gas, followed by either ether or chloroform, has the advantage of getting the patient anesthetised quickly with a minimum of anesthetic. An anesthetist who does not know how to administer all anesthetics has no place in an operating room, as a patient may be left in such a condition by the anesthetic, even if operation is skilfully performed, that the slightest amount of infection may prove serious. A carefully prepared patient, a rapid but careful operation, a competent anesthetist, and an exacting technic, make a happy combination in an operating room, which will give almost absolute assurance against infection.

1540 Main Street.

